# Bite-to-hospital time and morbidity in victims of viper bite in a rural hospital in Nigeria

**DOI:** 10.4102/phcfm.v4i1.371

**Published:** 2012-06-13

**Authors:** Oluwagbenga Ogunfowokan

**Affiliations:** 1 Jos University Teaching Hospital, Jos, Nigeria; 2 Department of Family Medicine, National Hospital, Abuja, Nigeria

## Abstract

**Background:**

Mortality amongst in-hospital patients bitten by carpet viper in northern Nigeria has reduced, related to use of a monospecific ovine Fab snake antivenom. However, many victims survive with temporary or permanent morbidity.

**Objectives:**

Study objectives were to: (1) determine and score the morbidity caused by carpet viper bite; and (2) find the relationship between bite-to-hospital time and morbidity amongst victims of carpet viper bite.

**Method:**

A prospective study was conducted in a rural hospital in north-central Nigeria. The morbidities scored were oedema, tenderness, prolonged whole-blood clotting time, blister, ulcer, need for blood transfusion, coma, hypotension, convulsion, length of hospital stay, need for disarticulation, and need for skin graft. A score of one was given to each objective sign. The bite-to-hospital time of 233 subjects was obtained. Descriptive and inferential statistical analysis was done.

**Results:**

Most of the subjects (150 or 64%) came to the hospital within 6 hours of the snake bite, with 2 (1%) arriving within 1 hour. The median bite-to-hospital time was 5 hours, with a range of 0.5–216 hours. Major morbidities were oedema, seen in 212 (91.0%; 95% CI 86.6–94.3%); incoagulable blood, seen in 205 (88%; 95% CI 83.1–91.9%), and tenderness, seen in 201 (86.3%; 95% CI 81.2–90.4%). The mean morbidity score was 8 ± 4. For every unit increase in logged bite-to-hospital time, the morbidity score increased by 1.85 (*p* < 0.001).

**Conclusion:**

Morbidity caused by carpet viper bite is high in Zamko, north-central Nigeria, and correlates with increasing bite-to-hospital time.

## Abstrait

**Temps écoulé entre une morsure et l'arrivée à l'hôpital et morbidité chez les victimes de morsures de vipères dans un hôpital rural au Nigeria**

**Contexte:**

La mortalité chez des patients hospitalisés victimes de morsures de vipères des pyramides dans le Nord du Nigeria a diminué grâce à l'utilisation d'un sérum antivenimeux FAB ovin monovalent. Cependant, nombreuses victimes survivent avec une morbidité temporaire ou permanente.

**Objectifs:**

Les objectifs de l’étude consistaient à: (1) déterminer et Marquer serait plus précis; ou bien coter la morbidité causée par la morsure d'une vipère des pyramides et (2) trouver la relation entre le temps écoulé entre la morsure et l'arrivée à l'hôpital et la morbidité chez des victimes de morsures de vipères des pyramides.

**Méthode:**

Une étude prospective a été réalisée dans un hôpital rural dans le centre-nord du Nigeria. Les morbidités marquees ou cotées incluaient des œdèmes, une douleur, un temps de coagulation du sang prolongé, des bulles, des ulcères, la nécessité d'une transfusion sanguine, un coma, une hypotension, des convulsions, une longue durée du séjour à l'hôpital, la nécessité d'une désarticulation et la nécessité d'une greffe de peau. Une note de 1 a été attribuée pour chaque signe objectif. Le temps écoulé entre la morsure et l'arrivée à l'hôpital des 233 sujets a été obtenu. Une analyse descriptive et déductive a été réalisée.

**Résultats:**

La plupart des sujets (150 ou 64%) sont arrivés à l'hôpital dans les six heures suivant la morsure du serpent, deux (1%) étant arrivés dans l'heure. Le temps moyen écoulé entre la morsure et l'arrivée à l'hôpital était de cinq heures, avec une fourchette comprise entre 0,5 et 216 heures. Les principales morbidités étaient des œdèmes, observés chez 212 patients (91.0%; 95% CI 86.6–94.3%); un sang incoagulable, observé chez 205 patients (88%; 95% CI 83.1–91.9%), et une douleur observée chez 201 patients (86.3%; 95% CI 81.2–90.4%). La note de morbidité moyenne était de 8 ± 4. Pour chaque augmentation unitaire du temps écoulé entre la morsure et l'arrivée à l'hôpital enregistrée, la note relative à la morbidité augmentait de 1.85 (*p* < 0.001).

**Conclusion:**

La morbidité causée par une vipère des pyramides est élevée à Zamko, dans le centre nord du Nigeria, et est corrélée à l'augmentation du temps écoulé entre la morsure et l'arrivée à l'hôpital.

## Introduction

### Key focus

Envenomation resulting from snake bites is an important public health hazard in many regions, particularly in tropical and subtropical countries.^1, 2^ The burden of snake bite morbidity and mortality still has a great impact on the population and on health care systems, especially in Africa, Asia, Oceania, and Latin America.^3^

#### Background

The pattern of clinical presentation amongst patients bitten by the carpet viper (*Echis Ocellatus*) in Nigeria has been studied, but little or no work has been done on the magnitude of morbidity caused by such bites and the relationship between bite-to-hospital time and morbidity.

#### Trends

Worldwide, of the estimated 5 million people bitten by snakes each year, 100 000–125 000 die, and both bites and deaths disproportionately affect rural populations in resource-poor settings.^4, 5^ More than 200 000 cases of snake bite are reported in India each year and 35 000–50 000 of them are fatal.^6^ In Africa, for instance, the incidence of snake bites in the Benue Valley of north-eastern Nigeria was 497 per 100 000 people per year, with a mortality of 12.2%.^7^ *Echis* species (saw-scaled or carpet viper) cause the largest numbers of bites and fatalities in northern Africa.^8^ Carpet viper is responsible for more than 95% of the snake bites in northern Nigeria.^9, 10^

Fortunately the observed mortality amongst in-hospital patients bitten by carpet viper in northern Nigeria has drastically reduced, related to the use of a monospecific ovine Fab snake antivenom (Echitab™).^9^ However, many victims survive with temporary or permanent morbidity, mostly due to the tissue-damaging effects of snake venoms. Incoagulable blood with local and systemic bleeding, local oedema, pain, ulcer, anaemia and coma are some of the morbidities observed amongst patients bitten by *E. ocellatus*.^10^

#### Objectives

The objectives of this study were to determine and score the morbidity amongst victims of carpet viper bite, and to find the relationship between bite-to hospital-time and morbidity amongst the patients.

#### Contribution to field

To our knowledge morbidity score, although important in quantifying the degree of morbidity and assessing the quality of life amongst survivors of carpet viper bite, has not received enough attention in the literature. This study provides information that health workers and policy makers in rural communities can use to educate the community and reduce morbidity after carpet viper bite.

## Ethical considerations

Ethical approval for the study was granted by the Research Ethics Committee of Jos University Teaching Hospital.

### Potential benefits and hazards

The study did not pose any undue risk to the patients beyond the usual care of obtaining venous blood with sterile needle and syringe to determine blood coagulability. The patients benefited by receiving free snake antivenom if the clotting time was prolonged. Study data were kept confidential.

### Recruitment procedures

Participation in the study was voluntary. Patients were also free to withdraw from the study at any time without affecting the usual care they would receive.

### Informed consent

Written informed signed consent was provided by the patients or their care-givers and given to the investigator.

### Data protection

Data were recorded in a case report form and kept secured. An electronic version of the data was also kept on the author's computer, that could only be accessed by the Ethics Committee on request.

## Trustworthiness

### Reliability

The morbidities observed were objective and verifiable and are consistent with those found in victims of carpet viper bite all over the world. The technique of measuring whole-blood clotting time used in the study is similar to that used by other workers and could be reproduced.

### Validity

Efforts were made to assure internal validity by including only morbidities that could be objectively assessed and taking the average of the bite-to-hospital time as reported by at least two witnesses. However, the score given to the objective signs which constituted the morbidity would need validation by other studies.

## Methods

### Materials

All confirmed carpet viper bite patients presenting consecutively to the casualty unit of Zamko Comprehensive Health Centre and who gave informed consent were enrolled. Confirmation of snake bite was by identification of the dead snake brought in by the patient or care-givers as *Echis ocellatus* by the investigators. All patients who claimed to have been bitten by a snake but neither saw the snake nor came with the carcass of the snake were excluded from the study. Also excluded were patients who could not tell the time they were bitten, as were their witnesses who could tell the time of the incident. Those patients whose information about the time they were bitten by a carpet viper varied by more than 2 hours from the time given by their care-givers were also excluded, in order to increase the precision of the bite-to-hospital time. Those who had received antivenom prior to presentation were also excluded from the study.

### Setting

The Jos University Teaching Hospital owns the Comprehensive Health Centre, a 35-bed primary and secondary health centre located in the rural community of Zamko in north-central Nigeria. Zamko is located on latitude 9°N, longitude 9.85°E, and has a savannah forest climate. Jos University Teaching Hospital is a tertiary hospital located in Jos town about 300 km from Zamko. The Comprehensive Health Centre had 8 050 patient visits in 2007, of which 2 031 (25.2%) were due to snake bite. Patients with snake bites accounted for 80.6% of all admissions. Snake-bitten victims came from both the local area and surrounding states, due to availability of free monospecific snake antivenom at the hospital. Most victims came to the hospital with the body of the snake.

### Design

This was a prospective study where sample size was determined using a standard formula^11^ for a study design to measure characteristics in terms of a proportion using 95% confidence level. The proportion of patients with morbidity due to envenomation after a bite by a carpet viper was 84.5% based on previous study^10^; a total expected confidence interval of 10% would require a minimum of 195 patients. Assuming a 20% attrition rate in from absconded patients or loss to follow-up, 235 patients were enrolled in the study.

### Procedure

On presentation all patients had 2 ml of venous blood withdrawn and put inside a glass tube^12^ for whole-blood clotting test by the method already described by Warrell et al.^13–15^ Those with non-clotting blood after 20 minutes were given 10 ml of monospecific ovine Fab snake antivenom by intravenous route. The time at which the patient was bitten by the snake was obtained from the patient and/or at least two other care-givers. If there was a disparity of 2 hours or less, the average time was recorded. All patients irrespective of age were admitted into male, female or paediatric snake-bite wards where only snake-bitten patients are managed.

The patients were examined and the whole-blood clotting test repeated every 6 hours.^12^ Intravenous injection of 10 ml monospecific ovine Fab antivenom was repeated if the venous blood of the patient remained non-clotting, or if there was reversal from clotting to non-clotting status. Persistence of normalised clotting time for three consecutive days and clinical fitness was ensured before patients with evidence of systemic envenomation were discharged from the hospital. Those with local but without systemic envenomation were discharged as soon as they were clinically fit. The victims who had neither systemic nor local envenomation were discharged after 24 hours. All discharged patients were told to come back in 2 weeks for follow-up or any time before the appointment day if they needed to. The follow-up appointment was to ensure persistence of normal clotting and clinical fitness. Trained community health workers, nurses, resident doctors and consultant family physicians were involved in the care of all snake-bitten patients.

The investigator recorded patient data on a case report form. A morbidity score developed by the investigator was computed to assess the extent of morbidity in each patient throughout the time of hospitalisation and follow-up. A score of one was given to each objective and verifiable sign. The same sign occurring in more than one area of the body attracted an additional unit score each. The morbidities scored were oedema, tenderness, blister, ulcer, need for blood transfusion, coma, hypotension, convulsion, angio-oedema, local bleeding, systemic bleeding, prolonged whole-blood clotting time on presentation, length of hospital stay, need for disarticulation, and need for skin graft. Each 10 ml of snake antivenom used attracted a unit score. Each day of admission attracted a unit score. Data from those who died were not included in the morbidity score, because death usually occurred early, resulting in a spuriously low morbidity score although the illness was fatal. The true morbidity score of those who absconded could not be determined, and hence they were not included in the final analysis. Only patients with a known morbidity end-point were analysed.

### Analysis

Data were analysed with Epi Info 3.3.2 (Centers for Disease Control, Atlanta Georgia). Results were expressed as mean and standard deviation for continuous normally distributed variables. The *t*-test was used to compare group means. Linear regression and correlations were used to assess relationships between continuous variables. Bartlett's test for homogeneity of variances was used to assess suitability of data for ANOVA. One-way ANOVA was used to compare means of several groups where appropriate. Qualitative data were compared using the Chi-squared test. Chi square for trend was used to assess the trend in the relationship between increasing levels of bite-to-hospital time and blood incoagulability on presentation to the hospital. A *p*-value of 0.05 or less was considered statistically significant.

## Results

### Demographic characteristics of patients

Two hundred and thirty five patients were enrolled. One patient died within 2 hours of presentation and another patient absconded after 2 days of admission. Of the 233 subjects who had a known morbidity end-point and whose data were analysed, 82 (35.2%) were female and 151 (64.8%) were male. The mean age of the patients was 23 ± 15 years (range 2–82). Children aged 12 years or less accounted for 70 (30%) of the subjects. One hundred and ninety three (82.8%) of the subjects were not residents of the local government area where the study was carried out. Most of the subjects were students, farmers and housewives, accounting for 89 (38.2%), 46 (19.7%), and 45 (19.3%) respectively. The usual places where snake bite incidents took place were homes, walking path, bush and farm, accounting for 71 (30.5%), 70 (30%), 48 (20.6%), and 42 (18.0%) respectively. The commonest sites of bite were the feet, accounting for 165 cases (70.8%). Most of the snake bite incidents occurred between 18h00 to 24h00, as shown in Table 1.

**TABLE 1:** Characteristics of patients bitten by carpet vipe.

Characteristic type	Characteristic	*n*	%
Age (yrs)	Mean 23; range 2-82; s.d. 15	-	-
Sex	Male	151	64.8
	Female	82	35.2
Location	Langtang LGA, Plateau State	40	17.2
	Other LGA, Plateau State	126	54.1
	Other States	67	28.7
Occupation	Students	89	38.2
	Farmers	46	19.7
	Housewives	45	19.3
	Others†	53	22.8
Place of bite	Home	71	30.5
	Bush path	70	30
	Bush	48	20.6
	Farm	42	18
	Others‡	2	0.9
Site of bite	Left foot	85	36.5
	Right foot	80	34.3
	Others§	68	29.2
Time of bite	06h01–12h00	32	13.7
	12h01–18h00	63	27
	18h01–24h00	119	51.1
	00h01–06h00	19	8.2

s.d., standard deviation; LGA, local government area.

† Civil servants, clergy, nomads, dependants, applicants.

‡ Market, school.

§ Left hand, right hand, abdomen, chest, face.

### Bite-to-hospital time

The median bite-to-hospital time was 5 hours (range, 0.5–216). The mean bite-to-hospital times of various groups of patients are shown in Table 2. Most of the subjects (150 or 64%) came to the hospital within 6 hours of the snake bite, as seen in Table 2. None of the independent variables were significantly associated with prolonged bite-to-hospital time.

**TABLE 2:** Morbidity score and bite-to-hospital time of various groups presenting with carpet viper bite.

Bite-to-hospital time group (hours)	*f*	%	Morbidity score			Bite-to-hospital time (hours)		
	
			Mean	s.d.	Range	Mean	s.d.	Range
< 1	2	1	7.5	0.7	7–8	0.6	0.1	0.5–0.7
1–6	148	63	7.6	3.4	1–17	3.7	1.5	1–6
> 6–144	81	35	9.1	3.4	1–23	27.8	34.6	7–144
144–216	2	1	8	1.4	7–9	202.5	19.1	189–216

s.d., standard deviation.

### Local and systemic envenomation

Of the 233 subjects who were analysed, 220 (94.4%; 95% CI = 90.6–97.0%) had local envenomation and 205 (88.0%; 95% CI = 83.1–91.9%) had systemic envenomation, as indicated by prolonged whole-blood clotting time. Evidence of systemic envenomation was present on arrival at the hospital in 192 subjects (82.4%; 95% CI = 76.9–87.1%). The median volume of antivenom administered was 10 ml (range, 10–70 ml).

### Morbidity

The mean morbidity score was 8 ± 4 (range 1–23). The mean morbidity score for the various groups according to bite-to-hospital time is shown in Table 2. The major morbidities were oedema, incoagulable blood, tenderness, and bleeding, accounting for 212 (91.0%; 95% CI 86.6–94.3%), 205 (88%; 95% CI 83.1–91.9%), 201 (86.3%; 95% CI 81.2–90.4%), 75 (32.2%; 95% CI 26.2–38.6%) respectively, as shown in Table 3. Of those who bled, bleeding occurred mainly from the site of bite and gingiva, accounting for 24 (32.0%) and 19 (25.3%) respectively. Sixteen (21.3%) had multiple bleeding sites. The mean duration of hospital stay was 3 ± 1.6 days (range 1–10).

**TABLE 3:** Morbidity amongst patients bitten by carpet viper (*n* = 233).

Clinical signs	*f*		95% CI

	*n*	%	
Oedema	212	91	86.6–94.3
Incoagulable blood	205	88	83.1–91.9
Tenderness	201	86.3	81.2–90.4
Bleeding†	75	32.2	26.2–38.6
Blister	20	8.6	4.0–12.5
Severe anaemia (transfused)	6	2.6	1.0–5.5
Hypotension	3	1.3	0.3–3.7
Coma	2	0.9	0.1–3.1
Others‡	6	2.6	1.0–5.5

*f*, Frequency; CI, Confidence interval.

† Includes gingivitis, epistaxis, haematuria, haematochexia, menorrhagia, sub-cojunctival haemorrhage, site of bite and old scar.

‡ Gangrene, ulcer, angio-oedema of the jaw, and convulsion.

### Correlation of bite-to-hospital time and morbidity score

**FIGURE 1:**
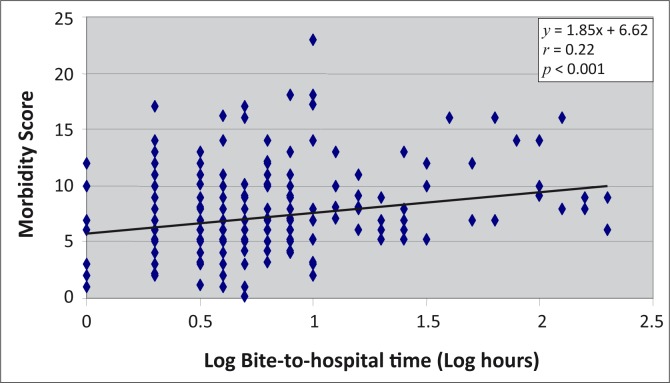
Relationship between morbidity score and logged bite-to-hospital time after carpet viper bite.

Logarithmic transformation of bite-to-hospital time was done because the distribution was skewed. Figure 1 shows the regression line describing the relationship between logged bite-to-hospital time and morbidity score. This relationship was significant (r = 0.22, *p* < 0.001), indicating a relationship between the extent of morbidity and the logged bite-to-hospital time amongst subjects bitten by carpet viper.

### Relationship between bite-to-hospital time and incoagulable blood at presentation

**FIGURE 2:**
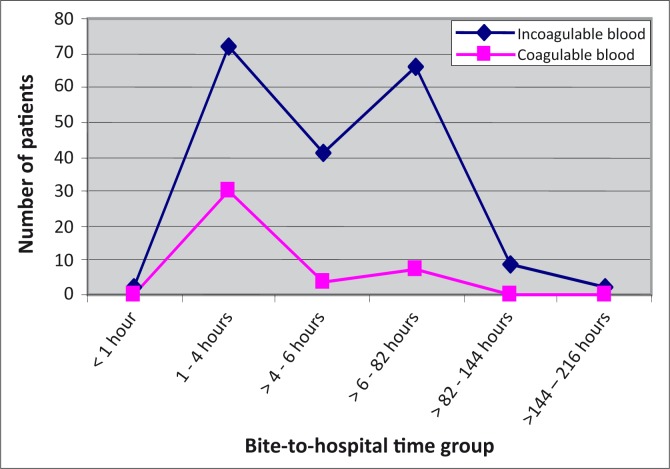
Proportion of patients with coagulable/incoagulable blood at presentation vis-a-vis bite-to-hospital time after carpet viper bite.

Figure 2 shows the proportion of subjects with whole-blood clotting time exceeding 20 minutes compared with those with normal whole-blood clotting time at presentation, according to bite-to-hospital time. There was a relationship between increasing bite-to-hospital time and blood incoagulability at presentation (Chi square for trend 4, *p* = 0.02).

### Relationship between morbidity and total dose of antivenom required

Dose of antivenom used was excluded from the computed morbidity score, and the relationship between resultant morbidity score and dose of antivenom required was then estimated. For every unit increase in morbidity score, the total dose of antivenom required increased by 2.5 ml in a linear relationship (r = 0.58, r^2^ = 0.34, *p* < 0.001). Hence there was a significant relationship between morbidity and total dose of snake antivenom required after carpet viper bite.

## Discussion

### Outline of results

The main aim of this study was to describe the extent of morbidity suffered by patients bitten by carpet viper and determine the relationship between morbidity and bite-to-hospital time. Only subjects with identifiable bite by carpet viper (*E. ocellatus*) were included in our study. Echis sp. is one of the snake species causing high morbidity and mortality globally.^8^ In rural Maharashtra in India, over a 10-year period *E. ocellatus* was found to account for 64.2% of venomous snake bites.^16^ A previous study showed that *E. ocellatus* caused 96% of identifiable cases of snake bite in our study location.^10^

The main finding was that the longer the bite-to-hospital time, the higher the extent of morbidity experienced by patients bitten by carpet viper, despite administration of monospecific antivenom which has drastically reduced the mortality. The overall morbidity and the major complication of incoagulable blood at presentation to the hospital were independently related to increasing bite-to-hospital time.

The median bite-to-hospital time of 5 hours found in our study was higher than those of 1 hour 26 minutes, 3 hours and 3.5 hours found by da Silva et al.,^17^ Kalantri and co-workers^6^ and Lalloo and associates.^18^ The higher median bite-to-hospital time in our study was probably due to the poor transportation system and bad roads. However, the median time in our study was less than the 7 hours 15 minutes and 9 hours found by Sloan and co-workers^19^ and Sharma and colleagues.^20^ Despite the relatively high median bite-to-hospital time in our study, 64% of the subjects presented to the hospital within 6 hours, in agreement with previous reports by other investigators.^10, 21^ Since 82.8% of the patients in our study came to the hospital from locations far away from the study area, and had poor means of transportation, many of them arrived fairly early. This finding suggested that once patients are aware of where they could receive affordable or free quality care, they do their best to access the care. Improved literacy level and good transportation systems, whilst improving the awareness level of populations at risk of snake bite, will further reduce bite-to-hospital time, as found by Suchithra, Pappachan and Sujathan^21^ Osmani and colleagues found that 73% of subjects presented late after snake bite because of initial delay resulting from prior visits to local practitioners, self-medication, long distance and transportation problems.^22^

Risk of developing complications such as renal failure and prolonged periods of incoagulable blood after initiating antivenom was found to be associated with late presentation to hospital after venomous snake bite, most by viper.^21, 23^ Other previous studies in India and Pakistan found greater complications, such as the need for blood transfusion, requirement for higher volumes of antivenom, longer duration of hospital stay, multiple bleeding sites, need for surgical intervention, development of local sepsis and permanent disabilities in subjects presenting late after viper bites.^22, 24, 25^ Our findings showed that in our locality, delay in presentation to hospital is associated with increasing morbidity, in agreement with what other investigators have found in other parts of the world.

In spite of the high morbidity, only one subject died, resulting in 0.4% mortality in this study. An earlier retrospective study by Madaki et al.^10^ found no mortality. Another done in north-eastern Nigeria found a mortality of 12.2%.^7^ The much lower mortality found in our study is most likely due to: 1) the availability of free monospecific ovine Fab snake antivenom raised against *E. ocellatus* found in our locality; 2) community awareness of the availability of the antivenom, which made many of the victims of snake bite access health care fairly early; and 3) a well-developed protocol of management of snake bite patients by an experienced and trained health care team in our study location.

The author also found that bite by carpet viper is an occupational hazard occurring mainly amongst young male farmers in the rural community. Similar findings were reported by other workers.^6, 10, 16, 26^ Many of the students in our study also carry out farming activities and hunt for bush rats. Such farming and hunting activities put them at risk of snake bite, since most snake bite incidents occurred not in the school but in the farm, bush and along bush paths. The mean age of 23 years found in our study is similar to that of 26 years found by Omogbai and colleagues^26^ in Benin City, Nigeria. This finding shows that the young, active males in the rural community are at highest risk, perhaps because of their adventure and tendency not to take precautions to prevent snake bite.

### Practical implications

The observed mortality amongst in-hospital patients bitten by carpet viper in north-central Nigeria has drastically reduced, related to use of a monospecific ovine Fab snake antivenom. However, many victims survive with temporary or permanent morbidity. The implication of the results of this study is that delay in presentation to hospital after carpet viper bite is related to the degree of morbidity suffered by the victims, despite the use of antivenom. Therefore, beyond making snake antivenom available and accessible, all stakeholders should ensure that victims are brought to the hospital as soon as possible to reduce the morbidity they suffer.

### Limitations of the study

The major limitation of this study is its observational nature. The author does not know the number of people bitten by snakes that did not come to the hospital or went elsewhere. It is possible that those with more severe envenomation were willing to travel a greater distance for care. This would cause a selection bias that could explain our findings that bite-to-hospital time were associated with more severe symptoms.

Another limitation relates to the nature of the morbidity score. Equal weighting given to each sign may not accurately measure the severity of morbidity. There is a need for more studies to validate the morbidity score.

### Recommendations

Preventive measures to protect the feet of populations at risk of snake bite, community education about the morbidity caused by carpet viper bite, and taking medical care of snake bite closer to those at risk, whilst sustaining availability of potent antivenom, may help reduce morbidities caused by carpet viper bite.

## Conclusions

Despite low mortality amongst patients bitten by carpet viper due to effective use of new monospecific ovine Fab snake antivenom, the morbidity level is still high and correlates with increasing bite-to-hospital time. It is important to reduce the bite-to-hospital time and morbidity amongst victims of carpet viper bite so that they can have a better quality of life. There is a need for other studies to validate the morbidity score used in this study.
